# Genome sequencing and analysis of *Salmonella enterica* serovar Typhi strain CR0063 representing a carrier individual during an outbreak of typhoid fever in Kelantan, Malaysia

**DOI:** 10.1186/1757-4749-4-20

**Published:** 2012-12-13

**Authors:** Ramani Baddam, Narender Kumar, Sabiha Shaik, Tiruvayipati Suma, Soo Tein Ngoi, Kwai-Lin Thong, Niyaz Ahmed

**Affiliations:** 1 Pathogen Biology Laboratory, Department of Biotechnology and Bioinformatics, School of Life Sciences, University of Hyderabad, Hyderabad, India; 2 Institute of Biological sciences, Faculty of Science, University of Malaya, Kuala Lumpur, Malaysia; 3 Laboratory of Biomedical Science and Molecular Microbiology, UMBIO Research Cluster, University of Malaya, Kuala Lumpur, Malaysia

## Abstract

*Salmonella* Typhi is a human restricted pathogen with a significant number of individuals as asymptomatic carriers of the bacterium. *Salmonella* infection can be effectively controlled if a reliable method for identification of these carriers is developed. In this context, the availability of whole genomes of carrier strains through high- throughput sequencing and further downstream analysis by comparative genomics approaches is very promising. Herein we describe the genome sequence of a *Salmonella* Typhi isolate representing an asymptomatic carrier individual during a prolonged outbreak of typhoid fever in Kelantan, Malaysia. Putative genomic coordinates relevant in pathogenesis and persistence of this carrier strain are identified and discussed.

## Background

*Salmonella enterica* serovar Typhi, the aetiologic agent of typhoid fever is still posing a major health problem for the developing world, as about 16 million new cases are reported each year [1]. *S*. Typhi causes systemic infections (typhoid fever) as well as chronic infections (asymptomatic carriers) in humans, the latter serve as the source of infection [2]. The transmission of *S*. Typhi is primarily through faecal-oral route and a significant number of infected individuals become chronic asymptomatic carriers and keep shedding *S*. Typhi in faeces for decades [3]. This results in endemicity of *S*. Typhi in regions of the world with underdeveloped sanitation and community hygiene [4].

Carrier identification becomes extremely important as some of the ancestral haplotypes were observed in recent isolates suggesting their persistence in these asymptomatic carriers [5]. Traditional methods such as culturing of bacteria from faecal samples are not fool proof as the carriers shed bacteria intermittently. Serological tests to detect specific antibodies such as anti-H and anti-O are unable to differentiate between carriers and individuals who have recovered from the infection [6]. Especially, in areas endemic for *S*. Typhi, due to high background levels of these antibodies, serological tests cannot be adopted for the identification of a carrier [7]. Thus, there is an urgent need for inexpensive and efficient detection methods for the establishment of carrier state, perhaps based on genomic markers.

The genetic typing tools such as PFGE, AFLP, ribotyping etc. can resolve limited genetic variation occurring within specific sites, and therefore are incapable of differentiating highly clonal strains such as outbreak related strains from the ones not associated with the outbreak (carrier isolates) [8-10]. High-throughput sequencing technologies have already been employed as a high resolution molecular epidemiologic tool to discern microevolution of highly related strains [11].

In this study, we attempted to determine if whole genome sequencing of *S*. Typhi isolated from a carrier individual can provide insights related to persistence and or adaptation mechanisms. We describe the genome sequence of a *Salmonella enterica* serovar Typhi strain (ST CR0063) isolated from a carrier individual during a prolonged outbreak of typhoid fever in Kelantan, Malaysia.

## Results and discussion

### Genome statistics

**Figure 1:**
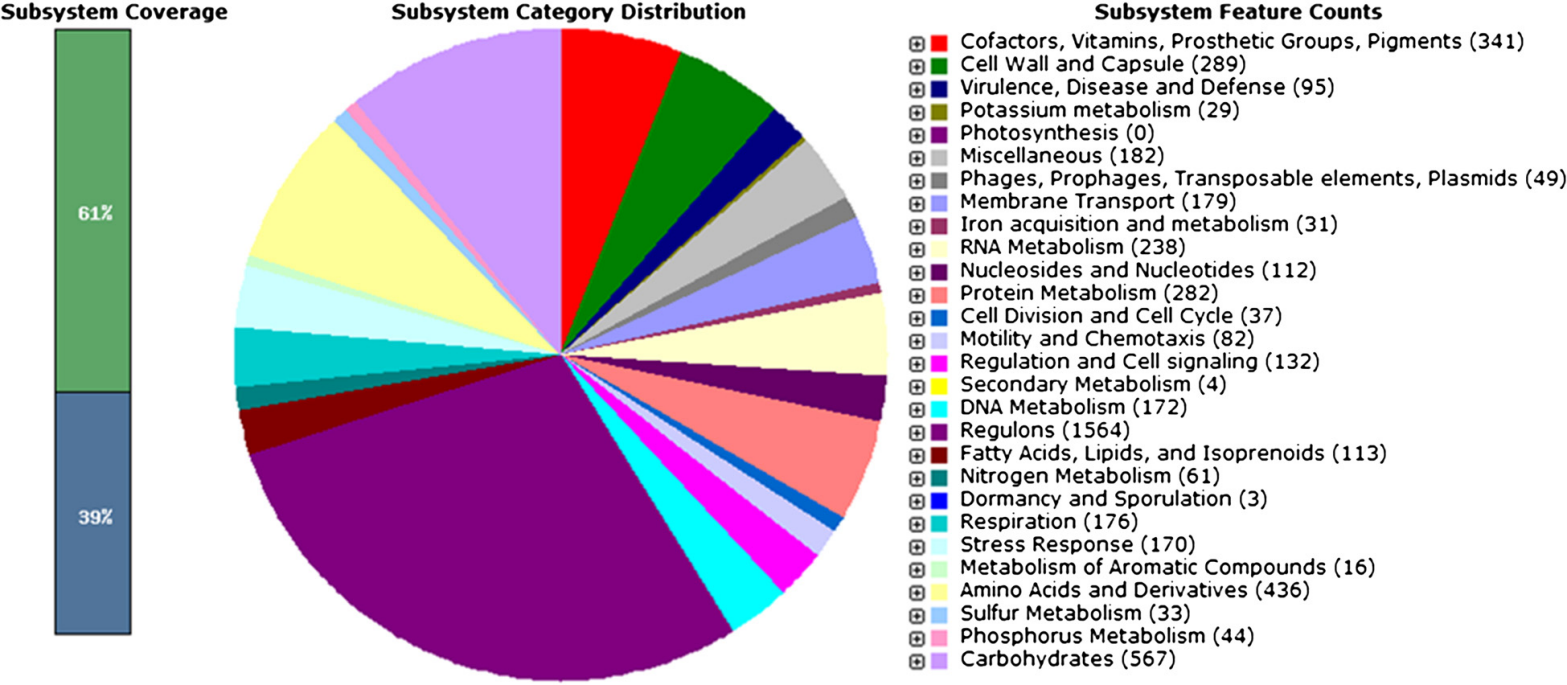
**Subsystem distribution of ST CR0063.** The subsystem statistics of ST CR0063 based on genome annotations performed according to RAST conventions.

**Figure 2:**
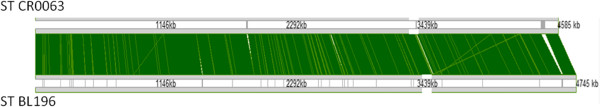
**Comparison of *Salmonella *Typhi strains ST CR0063 and ST BL196.** Comparison of whole genome sequences of *S*. *Typhi* strains using MG-CAT – one strain was isolated from a carrier individual (ST CR0063) and another from an infected individual (ST BL196) during a prolonged outbreak of Typhoid fever in Kelantan [13].

The size of the draft genome of *Salmonella* Typhi (ST CR0063) is 4,585,851 bp with a coding percentage of 86.1%. The G + C content of this strain is about 51.71%. The total number of CDS determined are 4946 with an average length of gene about 798 nucleotides. The genome of ST CR0063 revealed 77 tRNA and 22 rRNA genes. The subsystems distribution of basic metabolic machinery of this strain is represented in Figure 1. The assembled draft genome shows high degree of similarity and shared core genome regions with *Salmonella* Typhi ST BL196 [12], the one identified as associated with a typhoid outbreak in Kelantan during the same period (Figure 2).

### Virulence factors

The gene *shdA*, a key factor predicted to be involved in persistence of the bacterium in the intestines [14] by binding to its extracellular matrix, was identified and annotated. This gene, by mimicking the host heparin, is able to bind to the extracellular matrix proteins, fibronectin and collagen, and probably plays an important role in carriers by contributing to prolonged faecal shedding [15]. The *fim* gene cluster [16] of chaperone –usher family involved in adhesion to non-phagocytic cells was detected along with its negative regulator fimW. Type IV pili and *agf* operon [17,18] encoding curli fimbriae which aid in attachment of the bacterium to intestinal villi and also with each other, were found in the genome. These adherence factors determine the sites of bacterial colonisation and thereby adaptation and pathogenicity of a particular strain [19,20].

**Figure 3:**
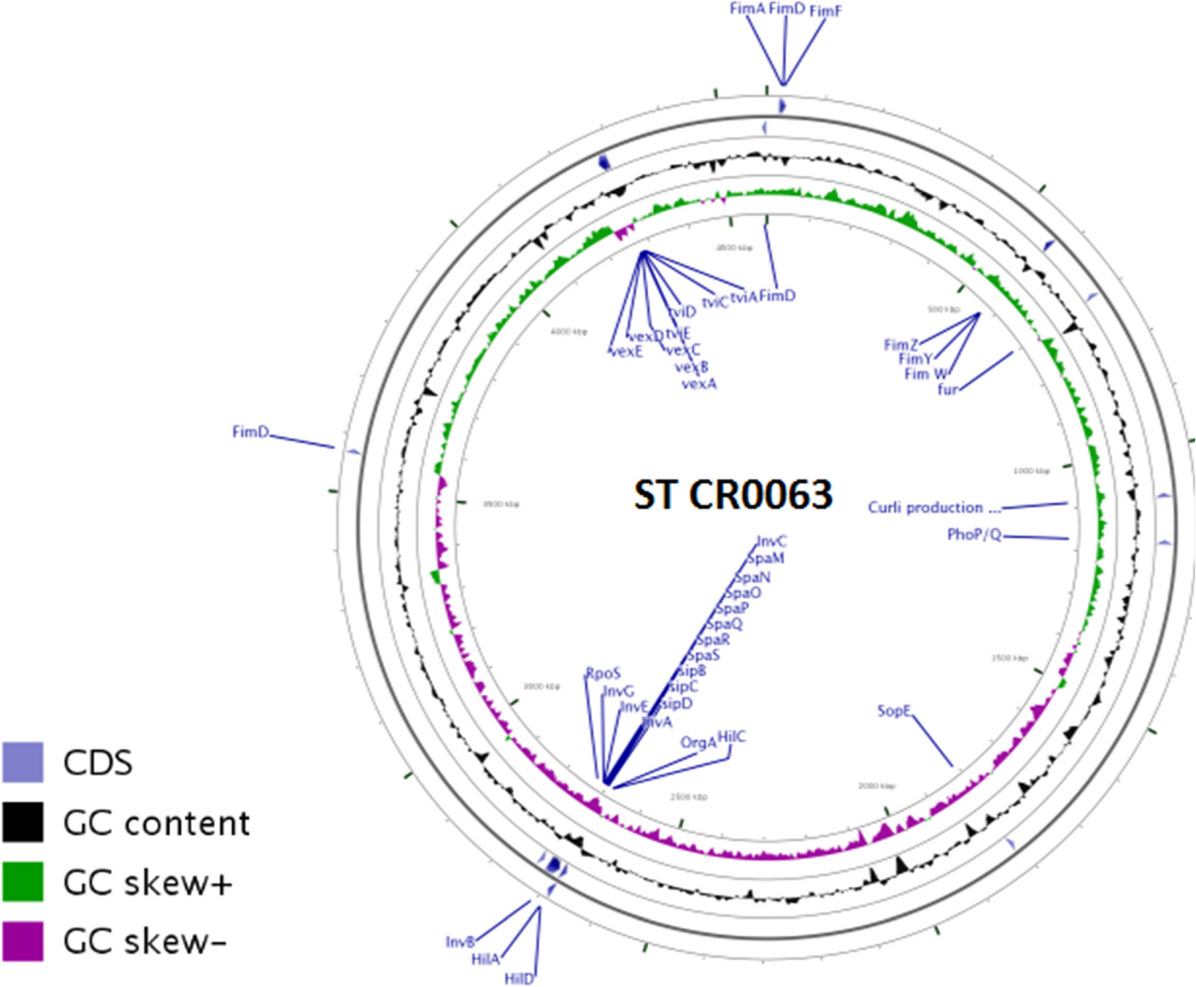
**Circular Genome view of ST CR063.** Positions of some of the major virulence factors and their regulators identified in ST CR0063 marked in the circular genome generated using CGview [26].

The *S*. Typhi strain ST CR0063 genome also revealed *viaA* and *viaB* loci, the prime regulators of Vi antigen expression. The *viaB* locus contains all genes for the biosynthesis (*tviAE*) and export (*vexAE*) of the Vi antigen, a well-known virulence factor [21,22]. The *mgtC* gene involved in Magnesium uptake and ferric uptake regulators (fur) [23] were also identified in ST CR0063. The PhoPQ regulon [24], which induces cytokine secretion and cationic antimicrobial peptide resistance, was also found to be conserved in our carrier strain. The RpoS sigma factor needed to cope up with external stress and nutrient depletion conditions [25] was also identified and annotated. The co-ordinates of these virulence factors in the genome of ST CR0063 are depicted in Figure 3.

### Phages and pathogenicity islands (PAIs)

The phages gifsy-1 and fels-2 [27] together with many phage proteins and a few hypothetical proteins were identified in the genome of ST CR0063 by various algorithms (See Methods for details). It is expected that these phages are acquired by horizontal gene transfer (HGT) events as they were embedded in some of the genomic islands recognized. The phage encoding SopE effector protein of SPI-1 (Salmonella Pathogenicity Island) was present in ST CR0063 as recognized in other Typhi genomes [28,29].

More than 15 PAIs that encode clusters of virulence associated genes have been identified across various serovars of *Salmonella enterica*. Ten pathogenicity islands have been identified by us in ST CR0063 and as expected [30], they were characterised by different G + C content and bounded by t-RNA genes. The SPI-1 type III secretion system (TTSS) structural genes spaMNOPQRS and invABCEFGH and their regulatory proteins HilA, HilC, HilD [31] were also identified and annotated. The SPI-1 secreted effector proteins SopE, SopE2, SipA, SipB, SipC and SptP required for endothelial uptake and invasion [32] are also present. The genes SpiC, SseF, SseG, SifA, SifB secreted by SPI-2 TTSS and that are needed for survival in macrophages and colonisation of host organs [33] were also recognised in the present genome. The known regulators of SPI-2, OmpR-EnvZ and PhoP-PhoQ [34] were present. SPI-3, identified by us, contained magnesium transport genes *mgtC* and *marT* which are required for survival in macrophages [35]. Type I secretion system and its associated proteins encoded by SPI-4, and that are involved in the invasion of the intestinal epithelium [36], were also located in the present genome. The SPI-1 effector proteins SopB and PipB associated with enteritis and coded by SPI-5 [37] were also detected and annotated. The chaperone-usher fimbrial operons carried by SPI-6, SPI-10 and bacteriocin immunity proteins carried by SPI-8 [38] were identified. The SPI-7 and SPI-9 were identified in the ST CR0063 genome and were found to encode *viaB* locus, type IV pili formation proteins and TISS [38,39].

### Conclusions and prospective

The genomic blueprint of *Salmonella* Typhi isolate ST CR0063 was elucidated in this study. The genome sequence information presented herein may be harnessed to guide comparative genomics and identification of novel and specific diagnostic markers. However, further studies involving large scale genome sequencing of the strains from several of the endemic countries and especially those from carrier individuals of different socio-economical settings is needed to develop a reliable approach to decipher the characteristics of a carrier state. Also, it will be required to determine the true extent of the diversity of carrier strains as juxtaposed to their acutely pathogenic forms in terms of 1) gene gain/loss during colonization and adaptation; 2) dynamics of virulence acquisition/attenuation; 3) possible genomic rearrangements; and 4) the relative preponderance of carrier and virulent strains circulating in different endemic regions of the world. Finally, an in-depth analysis of the host-pathogen interactions and their influence on gut microbiota can only explain the adaptation and persistence mechanisms of the (asymptomatic) carrier strains.

## Methods

### Genome sequencing

DNA was isolated from the stool sample of an asymptomatic carrier individual from Kelantan, Malaysia in 2007 during a prolonged outbreak. The draft genome sequence of this strain (STCR0063) was determined by Illumina Genome Analyzer (GAIIx, pipe- line ver l.6). The 100 bp paired-end sequencing was done with an insert size of 300 bp. About 67X genome coverage was achieved and 1.9 gigabytes of data were obtained.

### Assembly and annotation

The sequence data were assembled *denovo* in the same way as described previously [40-45] into 538 contigs using Velvet [46] at optimal hash length 39. SSPACE [47] was used for scaffolding the pre-assembled contigs using paired-end data. The gaps within these scaffolds were filled using Gapfiller by aligning the reads against already generated Scaffolds by SSPACE [48].

A reference guided assembly was generated by aligning reads to *Salmonella* Typhi str. CT18 [GenBank: AL513382.1] using bwa tools [49]. This reference guided assembly was used to re-order the scaffolds generated in de-novo way. In-house written Perl scripts were used for this re-ordering process and to finalize the gaps. The de novo and reference guided approaches were used to finalize the consensus draft genome. The reference guided assembly and reordered scaffolds were loaded on to Tablet – NGS data visualisation tool, to visualise the repeats, insertions and deletions [50].

The final draft nucleotide sequence after manual curation was annotated in our laboratory using RAST [51] and ISGA pipeline [52]. The genome statistics were gleaned using Artemis [53]. The data were further validated using gene prediction tools such as Glimmer [54] and EasyGene [55]. The RNAmmer [56] and tRNAscan-SE [57] were used to identify rRNA and tRNA respectively.

### Phages and PAIs

Prophages and putative phage like elements in the genome were identified using PhiSpy [58] and Prophage Finder [59]. The putative HGT events were determined using Alien Hunter tool [60]. An integrated interface Island Viewer was used to predict putative genomic islands within the genome [61].

### Sequence data access

The Salmonella enterica subsp. enterica serovar Typhi str. CR0063 whole genome shotgun (WGS) project has been submitted to the GenBank and has the project accession AKIC00000000. The project version entailing draft assembly described herein has the accession number AKIC01000000, and consists of sequences AKIC01000001-AKIC01000538.

## Competing interests

The authors declare that they have no competing interests.

## Authors’ contributions

NA designed the study, interpreted the results and edited the manuscript. RB and NK managed Illumina sequencing, made the assemblies, analyzed the genome, and performed annotations. SS and TS provided computational tools and contributed to automation of the analysis process. KT provided inputs related to the outbreak and the strain features, characterized the strain and maintained it in pure cultures. STN contributed to microbiology of the strain and prepared high molecular weight DNA for genome sequencing. All the authors read and approved the manuscript prior to submission.
